# Integrated multi‐month dispensing for HIV and hypertension in South Africa: A model of epidemiological impact and cost‐effectiveness

**DOI:** 10.1002/jia2.26413

**Published:** 2025-02-12

**Authors:** Youngji Jo, Sydney Rosen, Brooke E. Nichols, Lise Jamieson, Nkgomeleng Lekodeba, Robert Horsburgh

**Affiliations:** ^1^ Department of Public Health Sciences School of Medicine University of Connecticut Farmington Connecticut USA; ^2^ Department of Global Health Boston University School of Public Health Boston Massachusetts USA; ^3^ Department of Medical Microbiology Amsterdam University Medical Center Amsterdam the Netherlands; ^4^ Health Economics and Epidemiology Research Office, Faculty of Health Sciences University of the Witwatersrand Johannesburg South Africa; ^5^ The South African Department of Science and Innovation/National Research Foundation Centre of Excellence in Epidemiological Modelling and Analysis (SACEMA) Stellenbosch University Stellenbosch South Africa

**Keywords:** cost‐effectiveness, HIV, hypertension, integrated service delivery, modelling, multi‐month dispensing

## Abstract

**Introduction:**

In the current era of universal antiretroviral treatment (ART), health systems have the dual challenge of a growing number of people living with HIV and on ART who are also receiving chronic, life‐long treatment for non‐communicable diseases. Current evidence suggests that 6‐month multi‐month dispensing (6MMD) can maintain at least equivalent clinical outcomes to conventional care and reduce costs, but little is known when integrating 6MMD for multiple conditions. We examined the cost‐effectiveness of integrated multi‐month drug dispensing for people living with HIV and hypertension.

**Methods:**

Using an age‐ and sex‐specific hybrid decision tree and Markov state‐transition model, we constructed a 100,000‐person simulated population cohort who may develop HIV and hypertension and initiate treatment at clinics in South Africa over a 10‐year time horizon. We assessed the incremental costs and effectiveness of 6MMD versus conventional care from a health system perspective under different conditions of care‐seeking, eligibility and uptake of 6MMD for clinically stable patients. Model inputs were sourced from previously published literature. 6MMD was defined as reducing the frequency of clinic visits by increasing the number of medications dispensed to stable patients at each visit from 3 to 6 months. For the integrated 6MMD, we assumed that comorbid patients receive both HIV and hypertension drugs at the same facility on the same day.

**Results:**

Our study demonstrates that integrated 6MMD for HIV and hypertension in South Africa can avert between 0.8 and 1 DALYs and increase health systems costs between $24 and $49 per patient per year, compared to the status quo. One‐way sensitivity analysis showed that HTN drug cost and prevalence of HIVHTN and HIV were key drivers in the cost per DALYs averted. Overall, integrated 6MMD with a greater proportion of well‐controlled patients and lower mortality rates led to greater cost savings or better cost‐effectiveness (less than $50 per DALY averted) across a wide range of loss‐to‐follow‐up (LTFU) factor variation.

**Conclusions:**

By better controlling disease among patients already in care, integrated 6MMD can be more beneficial than the status quo treatment by resulting in fewer cases of LTFU and fewer deaths through high‐quality care.

## 1 INTRODUCTION

In the current era of universal antiretroviral treatment (ART), health systems face the challenge of managing a growing number of people living with HIV who also require chronic, life‐long treatment for non‐communicable diseases (NCDs) [1]. South Africa, home to the largest HIV epidemic globally, has 7.8 million people living with HIV as of 2022, with an adult prevalence of 18% [2]. A community‐based surveillance study in KwaZulu‐Natal found 34.2% had HIV, and 23.0% had elevated blood pressure [3]. Hypertension, a major cardiovascular risk factor among HIV‐positive individuals, has a prevalence of 5−55% in high‐income and 9−46% in low‐ and middle‐income countries [4, 5]. In 2017, hypertensive disorders accounted for 30% of deaths related to increased blood pressure in South Africa [6]. Despite national guidelines for hypertension prevention and treatment [7], uncontrolled hypertension (13.5−75.5%) is on the rise, partially because 85% of South Africans rely on public clinics, which face significant resource and staffing shortages [8, 9].

An increased number of patients on ART and NCD treatment contributes to clinic crowding, long waiting times and reduced care quality, especially in high‐density areas [10, 11]. Multi‐month dispensing (MMD) of medications, a key aspect of differentiated service delivery (DSD), aims to address these challenges by simplifying care, reducing unnecessary burdens on patients and the health system, and improving retention [12]. As countries scale up DSD for ART [13], the World Health Organization has recommended integrating NCD treatment into these programmes [14]. Well‐organized, integrated MMD could, in theory, reduce clinic visits for stable patients, mitigating crowding, improving efficiency and enabling providers to focus on high‐risk patients needing more attention, such as those who have recently initiated treatment or are clinically unstable for HIV or other conditions [15, 16, 17].

In 2014, South Africa's National Department of Health initiated the Central Chronic Medicines Dispensing and Distribution (CCMDD) programme to improve ART adherence and retention. This programme allows patients to collect medications from convenient community‐based locations, reducing travel costs, wait times and clinic congestion [18, 19]. Initially targeting stable HIV and NCD patients, the programme served as a strategy for managing repeat prescriptions and expanded the initial 11 pilot districts for national coverage in 2016 [20]. Despite CCMDD's benefits, evidence supporting integrated HIV and NCD care in sub‐Saharan Africa mainly comes from facility‐based cross‐sectional studies with relatively small samples and many undiagnosed NCD cases [21, 22]. Previous studies show that 6‐monthly MMD (6MMD) for HIV maintains equivalent clinical outcomes to conventional care (i.e. monthly or 3‐monthly dispensing) while reducing costs [23], but there is relatively little evidence on treatment retention and cost‐effectiveness when integrating MMD for multiple conditions [24, 25]. Using a Markov state transition model, we examined the cost‐effectiveness of various hypothetical scenarios of integrated 6MMD for both HIV and hypertension, assuming improved clinical outcomes.

## 2 METHODS

### 2.1 Overview

We developed a hybrid decision tree and Markov state‐transition model to estimate service volume and health outcomes in the care cascade and identify the potential benefits and costs of an integrated multi‐month medication dispensing strategy in South Africa. The model assumed a 100,000‐person simulated cohort of a general population in which aged 14 years and older individuals may have or develop HIV or HIV and hypertension comorbidity (HIVHTN) and initiate HIV and/or HTN treatment at clinics in a South African setting as a reference point for this analysis [26]. The baseline age‐ and sex‐based prevalence of HIV [3] and hypertension [27, 28] in the population were sourced from South Africa's population‐based survey in South Africa. Model outcomes were estimated by following the cohort over 10 subsequent years (2022−2031).

The primary health outcome was evaluated as disability‐adjusted life years (DALY) averted. DALYs were calculated by assigning disability weights [29] to the time spent in each Markov state (with disutility experienced due to both HIV and hypertension with on and off treatment condition) to calculate years of life with disability (YLD) and adding this to years of life lost (YLL), calculated as the discounted life expectancy [30] at the time of death for individuals who die during the 10‐year time horizon regardless of cause. YLL beyond the 10‐year horizon is not included, as a conservative assumption given the uncertainty of events (e.g. innovations in NCD or HIV management) that may occur beyond that time horizon. DALYs are averted through the effects on the total number of patients within and outside of health systems (i.e. different disutility rates under on/off treatment) that result from care seeking/re‐engagement levels of patients, reduced loss‐to‐follow‐up (LTFU) and decreased mortality of stable patients with 6MMD compared with status quo without 6MMD. We also reported the number of stable (controlled) patients gained and the number of deaths averted as secondary outcomes. Deaths were counted by the absolute total number of people who died within and outside of the health systems over the 10‐year horizon. Model costs were evaluated from a health system perspective. These included the unit cost of outpatient visits, the monthly drug cost of ART and the monthly drug cost of hypertension preventive treatment in South Africa based on published references [31, 32, 33]. Costs and effects were both discounted at a rate of 3% per year. Our primary outcome was the incremental cost‐effectiveness ratio (ICER), expressed as 2022 US dollars per DALY averted. The primary model parameter values were obtained from published references collected from a PubMed search on 30 January 2024, which are provided in Table 1.

**Table 1:** Service delivery strategies

Strategies	Descriptions
Status quo (No 6MMD/ Integration)	All patients receive ART/HTN drug dispensing every 3 months without MMD and integration. All HIVHTN patients receive ART and HTN drugs in different venues on different visit days without integration. Health outcome (DALY/Deaths) including all people living with HIV and HIVHTN with different visit frequencies by disease condition: (1) Controlled HIV: 4 visits per year; (2) Uncontrolled HIV: 4 visits per year; (3) Controlled HIVHTN: 8 visits per year; (4) Uncontrolled HIVHTN: 8 visits per year.
6MMD for HIV only	Controlled HIV patients receive ART drug dispensing every 6 months with MMD but uncontrolled HIV or HIVHTN patients and stable HIVHTN receive ART drug dispensing every 3 months without MMD. All HIVHTN patients receive ART and HTN drugs in different venues on different visit days without integration. Health outcome (DALY/Deaths) including all people living with HIV and HIVHTN with different visit frequencies by disease condition and 20% reduced LTFU and mortality rates compared to the status quo for all those in care; (1) Controlled HIV: 2 visits per year; (2) Uncontrolled HIV: 4 visits per year; (3) Controlled HIVHTN: 4 visits per year; (4) Uncontrolled HIVHTN: 8 visits per year.
6MMD for HIV+HIVHTN	Controlled HIV and HIVHTN patients receive ART/HTN drug dispensing every 6 months with MMD but uncontrolled HIV or HIVHTN patients receive ART drug dispensing every 3 months without MMD. All HIVHTN patients receive ART and HTN drugs in different venues on different visit days without integration. Health outcomes (DALY/Deaths) include all people living with HIV and HIVHTN with different visit frequencies by disease condition and 20% reduced LTFU and mortality rates compared to the status quo for all those in care: (1) Controlled HIV: 2 visits per year; (2) Uncontrolled HIV: 4 visits per year; (3) Controlled HIVHTN: 4 visits per year; (4) Uncontrolled HIVHTN: 8 visits per year.
Integrated 6MMD for HIV+HIVHTN	Controlled HIV and HIVHTN patients receive ART/HTN drug dispensing every 6 months with MMD but uncontrolled HIV or HIVHTN patients receive ART drug dispensing every 3 months without MMD. All HIVHTN patients receive ART and HTN drugs in the same venues on the same visit days with integration. Health outcomes (DALY/Deaths) include all people living with HIV and HIVHTN with different visit frequencies by disease condition and 50% reduced LTFU and mortality rates compared to the status quo for all those in care: (1) Controlled HIV: 2 visits per year; (2) Uncontrolled HIV: 4 visits per year; (3) Controlled HIVHTN: 2 visits per year; (4) Uncontrolled HIVHTN: 8 visits per year.

The 6MMD in our model was defined as reducing the frequency of clinic visits from 4 to 2 per year for stable patients by increasing the number of medications dispensed to patients at each visit from 3 to 6 months. For the integrated (intervention) scenario, we assumed that comorbid patients received both HIV/HYP drugs at the same facility on the same day. Without coordination between ART care and hypertension care, the number of visits an individual may be expected to make to the healthcare facility could be as many as 8 per year without 6MMD and 4 per year with 6MMD. We compared this intervention to conventional care. Conventional care is defined as a 3‐month dispensing of ART at quarterly clinic visits in South Africa [34].

### 2.2 Model structure

**Figure 1:**
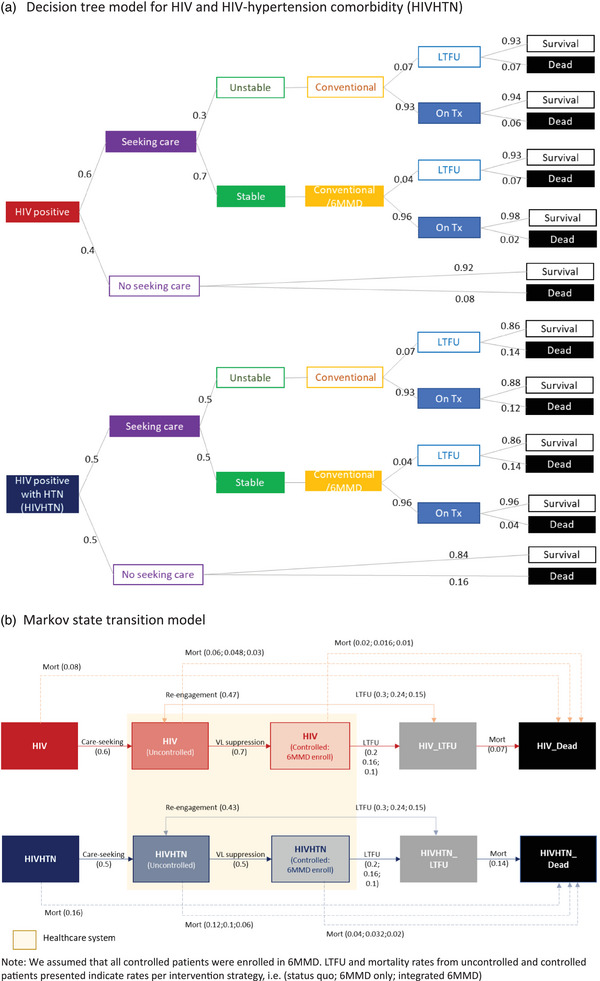
Conceptual framework. (A) Decision tree model for HIV and HIV‐hypertension comorbidity (HIVHTN). (B) Markov state transition model. *Note*: We assumed that all controlled patients were enrolled in 6MMD. LTFU and mortality rates from uncontrolled and controlled patients presented indicate rates per intervention strategy, that is (status quo; 6MMD only; integrated 6MMD).

Patients were characterized by disease status (HIV or HIVHTN), treatment initiation [35, 36], viral load suppression (uncontrolled/controlled) [37, 38], 6MMD enrolment, retention/attrition [39, 40] and survival/death [41] (Figure 1). For HIV and HIVHTN patients, we considered the probability of seeking care and not seeking care which determines the number of patients entering the health system [37, 42]. After seeking care and being on treatment for 6 months after treatment initiation, a proportion of patients were assumed to be well‐controlled (defined as viral suppression <1000 copies/ml for HIV patients and systolic blood pressure [SBP] <140 mmHg and diastolic blood pressure [DBP] <90 mmHg for persons with hypertension) and to be eligible to enrol in 6MMD [43, 44, 45]. Patients not well‐controlled on either HIV or HIVHTN before or after 6 months of treatment were assumed to receive conventional care (3‐monthly clinic visits). For both HIV and HIVHTN patients, we assumed higher annual LTFU rates among those who initiated treatment and were not well‐controlled for the first 6 months (0.3 LTFU rate) compared to those who were on treatment for 6 months and well‐controlled (0.2 LTFU rate) [35, 39, 40]. Similarly, we assumed higher annual mortality rates for those LTFU compared to those on treatment (0.07 vs. 0.06 with uncontrolled and 0.02 with controlled HIV patients; 0.14 vs. 0.12 with uncontrolled and 0.04 with controlled HIVHTN patients) [46]. The model also allows re‐engagement into treatment from those LTFU (at an annual probability of 0.47 for HIV patients and 0.43 for HIVHTN patients) (Table 1).

We assumed that the implementation of 6MMD and integrated service delivery for HIV and HTN could improve the health system service quality for all patients in care (by giving more time and resource capacity to provide care to more complicated cases), thus reducing LTFU and mortality for all patients. To reflect the health systems service quality, we assumed implementation of either 6MMD or integration alone to HIV or HIVHTN patients may reduce both LTFU and mortality by 20% (compared to LTFU/mortality in patients of the status quo without 6MMD/integration delivery) for all patients and implementation of integrated 6MMD (i.e. both integration and 6MMD) to all HIV and HIVHTN patients may reduce both LTFU and mortality up to 50% [35]. These estimates are based on the literature for integrated care; however, the impact of 6MMD on hypertension has not been shown to improve LTFU or mortality in previous studies [47]. For simplicity of analysis, we assumed patients were well‐controlled HIVHTN patients if both HIV and HTN conditions were stable (viral suppression under 1000 copies/ml and SBP/DBP under <140 and <90 mmHg) and they were eligible to enrol in 6MMD; however, if either HIV or HTN was not stable, we assumed those patients were HIVHTN uncontrolled patients.

### 2.3 Scenarios analysed

Given the uncertainty around the values of key parameters, we estimated incremental cost‐effectiveness across a range of scenarios (Table 2) under different values of four key factors: (1) care‐seeking level; (2) percentage of patients stable on ART after 6 months; and (3) adoption of integrated 6MMD for patients with HIV and HIVHTN. For each scenario, we set a base case reference scenario without any 6MMD integrated service delivery. The care‐seeking level (range 40−90% for HIV patients and 30−70% for HIVHTN patients) determines the number of patients entering the health system; the percentage of stable patients in care determines the number of patients in care who are eligible to enrol in 6MMD. Adoption of 6MMD determines the number of patients visiting health clinics every 3 or 6 months (4 or 2 visits per year). Finally, integration determines the number of comorbid patients visiting health clinics 4 or 8 visits per year with and without 6MMD. These translated into total health outcome and programme implementation costs for each scenario over 10 years (Table 2).

**Table 2:** Key input parameters

Parameter description	Model value		Reference
**Demographic parameters**	**Male**	**Female**	
Population			
Age 0–14	14,399 (30%)	13,900 (27%)	Estimated from age distribution based on a hypothetical 100,000 general population in South Africa [26]
Age 15–24	8063 (17%)	7799 (15%)	
Age 25–44	16,715 (34%)	16,469 (32%)	
Age 45–64	7622 (16%)	9180 (18%)	
Age 65+	1950 (4%)	3902 (8%)	
**Target population**	**Male**	**Female**	
HIV only			
Age 15–24	268 (10%)	487 (10%)	Sensitivity analyses varied the HIV initial prevalence ±20% [2, 3]
Age 25–44	2010 (72%)	3584 (72%)	
Age 45–64	480 (17%)	877 (18%)	
Age 65+	23 (1%)	58 (1%)	
HIVHTN			
Age 15–24	115 (5%)	325 (9%)	Sensitivity analyses varied the HIVHTN initial prevalence ±20% [28]
Age 25–44	1184 (53%)	2077 (58%)	
Age 45–64	880 (39%)	970 (27%)	
Age 65+	69 (3%)	231 (6%)	
**Transition parameters (annual probability)**			
HIV			
Probability of untreated HIV to death	0.08 (0.07−0.1)		Estimated average male survival years in the absence of ART in South Africa: 12.00 (10.12–14.04) [41]
Probability from HIV positive to HIV uncontrolled (care‐seeking/linkage to care)	0.6 (0.4−0.9)		Percentages of diagnosed, engaged in care (or not) and optimally treated (or not) for HIV patients in KwaZulu‐Natal, South Africa [45] Among living patients, 82% knew their HIV status, 45% were linked to care from a population‐based longitudinal HIV cascade‐of‐care study in KwaZulu‐Natal, South Africa [43] Among the overall study population, half were initiated on ART within 14 days after diagnosed (53.6%, 95% confidence interval [CI] 52.4–54.8) and three quarters were initiated within 60 days after diagnosed (75.5%, 95% CI 74.5–76.5). Overall, almost all of those diagnosed (98.1%) were eventually initiated on ART in South Africa [42]
Probability from HIV uncontrolled to HIV controlled (6MMD enrol)	0.7 (0.5−0.9)		Viral suppression rate 66% in South Africa [37, 38, 45]
Probability from HIV uncontrolled to LTFU^a^	Status quo: 0.3 (0.15–0.45) Any 6MMD/Integration: 0.24 (0.1–0.3) All 6MMD+Integration: 0.15 (0.1−0.2)		In South Africa, pre‐ART early retention and LTFU among PLHIV have been consistently estimated at 20−30% of patients [39] Meta‐analytic association between baseline CD4 count and LTFU shows that the risk of lost‐to‐follow up is higher in patients with low than high CD4 count [40] Pooled estimates of the increased rate of ART adherence by integrating HIV services and other health services (1.13−1.68) in sub‐Saharan Africa [35]
Probability from HIV controlled to LTFU^a^	Status quo: 0.2 (0.1–0.3) Any 6MMD/Integration: 0.16 (0.0.8–0.24) All 6MMD+Integration: 0.1 (0.05−0.15)		
Probability from HIV uncontrolled to death^b^	Status quo: 0.06 (0.05−0.09) Any 6MMD/Integration: 0.048 (0.04−0.07) All 6MMD+Integration:0.03 (0.025−0.045)		
Probability from HIV controlled to death^b^	Status quo: 0.02 (0.01–0.03) Any 6MMD/Integration: 0.016 (0.008−0.024) All 6MMD+Integration: 0.01 (0.005−0.015)		Estimated by observed 0–12 months mortality rates (per 100 person‐years) for men (8.65) and women (5.33) whose CD4 count is 50–199 cells/µl in South Africa [46]
Probability from HIV_LTFU to death	0.07 (0.035–0.105)		Estimated by observed >12 months mortality rates (per 100 person‐years) for men (2.11−2.65) and women (1.13−2.46) whose CD4 count is 50–199 cells/µl in South Africa [46]
Probability from HIV_LTFU to HIV uncontrolled (re‐engagement)	0.465 (0.23–0.7)		Assumed as an LTFU rate in between untreated HIV mortality (0.08) and uncontrolled HIV mortality (0.06)
HIVHTN			Estimated from (1−0.07)*0.5 and 0.07 as the HIV LTFU mortality, assuming that 50% of those who remain alive seek re‐engagement
Probability of untreated HIVHTN to death	0.16 (0.14−0.2)		Assumed as a two times higher mortality rate than the mortality of untreated HIV patients [41]
Probability from HIVHTN positive to HIVHTN uncontrolled (care seeking/linkage to care)	0.5 (0.4−0.8)		Fifty‐five percent of HIV patients had measured at least one of the three chronic diseases from a large, community‐based, cross‐sectional survey in KwaZulu‐Natal, South Africa [45]
Probability from HIVHTN uncontrolled to HIVHTN controlled (6MMD enrol)	0.5 (0.3−0.7)		Among hypertension patients who were aware of their hypertension status, control of hypertension was 47% in sub‐Saharan Africa [36, 45]
Probability from HIVHTN uncontrolled to LTFU^a^	Status quo: 0.3 (0.15–0.45) Any 6MMD/Integration: 0.24 (0.1–0.3) All 6MMD+Integration: 0.15 (0.1−0.2)		In South Africa, pre‐ART early retention and LTFU among PLHIV have been consistently estimated at 20−30% of patients [39] Meta‐analytic association between baseline CD4 count and LTFU shows that the risk of loss‐to‐follow‐up is higher in patients with low than high CD4 count [40] Pooled estimates of the increased rate of ART adherence by integrating HIV services and other health services (1.13−1.68) in sub‐Saharan Africa [35]
Probability from HIVHTN controlled to LTFU^a^	Status quo: 0.2 (0.1–0.3) Any 6MMD/Integration: 0.16 (0.0.8–0.24) All 6MMD+Integration: 0.1 (0.05−0.15)		
Probability from HIVHTN uncontrolled to death^b^	Status quo: 0.12 (0.1−0.18) 6MMD/Integration: 0.1 (0.08−0.14) All 6MMD+Integration: 0.06 (0.05−0.09)		Estimated from HIV uncontrolled mortality (0.06) and controlled mortality (0.02) and a large prospective study of adults living with HIV in Haiti that hypertension conferred a >2‐fold increased risk of death [49]
Probability from HIVHTN controlled to death^b^	Status quo: 0.04 (0.02−0.06) 6MMD/Integration: 0.032 (0.016−0.05) All 6MMD+Integration: 0.02 (0.01−0.03)		
Probability from HIVHTN LTFU to death (mortality)	0.14 (0.07−0.21)		Assumed LTFU rate in between untreated HIVHYP mortality (0.16) and uncontrolled HIVHYP mortality (0.12)
Probability from HIVHTN_LTFU to HIVHTN uncontrolled (re‐engagement)	0.43 (0.215−0.645)		Estimated from (1−0.14)*0.5 and 0.14 as HIVHYP LTFU mortality, assuming that 50% of those who remain alive seek re‐engagement
**Disability weight for DALYs**			
HIV on treatment	0.078 (0.052−0.111)		The disability of HIVHTN on (0.127) and off (0.679) treatment was estimated by adding hypertension disabilities (0.041 ∼ 0.049) to the disability of HIV on (0.078) and off (0.582) [29]
HIV off treatment	0.582 (0.406−0.743)		
HIVHTN on treatment	0.127 (0.083−0.183)		
HIVHTN off treatment	0.679 (0.471−0.882)		
Cost parameters			
ART cost per person per month	$13 ($12−$14)		Costs including ARV and non‐ARV drug cost and laboratory test cost in public clinics [31]
HTN drug cost per person per month	$14 ($2−$85)		Hypertension drug costs ranged from $2 to $85 per person‐month [32] Costs including antihypertensive treatment: thiazide, ACE inhibitor, calcium channel blocker, beta‐blocker, and annual electrolytes and urea labs in public clinics [33]
Outpatient cost per person per visit	$18 ($13−$23)		Outpatient costs for HIV care include clinical/non‐clinical staff costs and fixed costs in public clinics [31] Outpatient costs for HTN include physician and nurse visits in public clinics [33]

Abbreviations: ART, antiretroviral therapy; HIVHTN, HIV‐hypertension comorbidity; LTFU, loss‐to‐follow‐up; MMD, multi‐month drug dispensing.

For each scenario, we estimated ICERs of integrated 6MMD intervention for HIV and HIVHTN compared to the status quo (conventional care) under different conditions of care‐seeking (low/high: 40%/90% for HIV; 40%/80% for HIVHTN) and eligibility and enrolment of 6MMD (proportion of well‐controlled patients low/high: 50%/90% for HIV; low/high; 30%/70% for HIVHTN) for HIVHTN (Tables 1 and 2). We then performed one‐way sensitivity analyses of the integrated 6MMD strategy compared to the status quo to describe the association between each input variable in our model and the primary outcome (i.e. ICER) for each key population in each state. To better explore the simultaneous effect of uncertainty ranges across all of our model parameters, we also conducted a probabilistic sensitivity analysis, in which all model parameter values were randomly sampled over pre‐specified distributions (Appendix Table S1). This process was repeated 1000 times to generate uncertainty estimates around the primary ICER estimate, to be illustrated as the cost‐effectiveness plane and cost‐effectiveness acceptability curves (Appendix Figure S1). Finally, as it is uncertain whether and to what extent integrated 6MMD would reduce LTFU and mortality compared to the status quo [23], we also performed three‐way sensitivity analyses to quantify the impact of the varying LTFU and mortality rates (± 50%) by integrated MMD compared to the status quo on ICER by different care‐seeking proportions and different percentages of stable patients (Figure 3) [46, 48, 49].

### 2.4 Ethics

This modelling exercise is based on previously published literature and does not involve any human subjects research. Therefore, consent was not required for this study.

## 3 RESULTS

We estimated our target population (people living with HIV only and HIVHTN) consisted of 7787 HIV‐only patients (5006 female; 2781 male) and 5850 HIVHTN patients (3603 female; 2247 male) in 2022 based on a 100,000‐person standardized general population. In the base case scenario simulation (60% care‐seeking and 70% well‐controlled patients), 12,857 deaths and 451,815 discounted DALYs were generated and incurred $34 million of discounted total health systems costs with conventional care (status quo) over 10 years (Table 3). When 6MMD was applied to either (a) HIV patients only or (b) both HIV and HIVHTN patients under the same proportions of care‐seeking and well‐controlled patients, it was estimated there would be 1297 deaths averted and 42,421 DALYs averted in both scenarios compared to the status quo; these outcomes were the same because we assumed any 6MMD without integration would result in the same overall improvement of service quality to all patients (at 20% reduced LTFU and mortality compared to the status quo). These strategies, however, would incur incremental costs of $1.8 million in (a) and cost savings of $268,000 in (b), respectively, compared to the status quo; outpatient visit costs were substantially reduced when the 6MMD was implemented for (b) both HIV and HIVHTN simultaneously. Compared to 6MMD that was not integrated, implementing integrated 6MMD for HIV and HIVHTN averted approximately three times greater the number of deaths (3706 vs. 1297) and DALYs (120,653 vs. 42,421), cost an additional $4 million, and resulted in an ICER of $37 per DALY averted. The total health systems cost was expected to increase by about 13% compared to the status quo due to an increased number of patients in care with improved adherence (assuming a greater overall improvement of service quality to the patients in care, which we assumed would reduce LTFU and mortality by 50% from the status quo).

**Table 3:** Health and cost (2022 USD) outcomes per 100,000 general population over 10 years (2022−2031) by scenarios defined by care‐seeking and proportion of controlled patients

	Number of patients 2022–2031				Health outcome 2022–2031												
	Total patients in care		Total patients controlled		Total deaths		Total DALYs		Total cost 2022–2031		Incremental health outcome						
Service delivery strategy	*N*	%	*N*	%	*N*	%	*N*	%	2022 $USD (000)	%	Increased stable patients	Deaths averted	DALY averted	Incremental costs 2022 $USD (,000)	Incremental cost per additionally controlled patient	Incremental cost per death averted	Incremental cost per DALY averted
**Base case: Care seeking (HIV: 60% HIVHTN:50%); proportion of well‐controlled patients (HIV: 70% HIVHTN: 50%)**																	
Status quo	125,478	Ref	85,979	Ref	12,857	Ref	451,815	Ref	34,119	Ref	Ref	Ref	Ref	Ref	Ref	Ref	Ref
6MMD for HIV only	138,950	11%	99,512	16%	11,560	−10%	409,393	−9%	35,963	5%	13,533	1297	42,421	$1843	$136	$1421	$43
6MMD for HIV+HIVHTN	138,950	11%	99,512	16%	11,560	−10%	409,393	−9%	33,852	−1%	13,533	1297	42,421	−$268	−$20	−$206	−$6
Integrated 6MMD for HIV+HIVHTN	163,360	30%	125,345	46%	9151	−29%	331,162	−27%	38,560	13%	39,366	3706	120,653	$4441	$113	$1198	$37
**Scenario 1: Care seeking (low: HIV 40% HIVHTN 40%); proportion of well‐controlled patients (Low: HIV 50% HIVHTN 30%)**																	
Status quo	113,113	Ref	65,468	Ref	13,692	Ref	481,313	Ref	30,705	Ref	Ref	Ref	Ref	Ref	Ref	Ref	Ref
6MMD for HIV only	125,472	11%	76,569	17%	12,464	−9%	441,335	−8%	32,689	6%	11,101	1228	39,977	$1984	$179	$1616	$50
6MMD for HIV+HIVHTN	125,472	11%	76,569	17%	12,464	−9%	441,335	−8%	31,188	2%	11,101	1228	39,977	$483	$44	$393	$12
Integrated 6MMD for HIV+HIVHTN	148,166	31%	98,191	50%	10,144	−26%	366,414	−24%	36,029	17%	32,724	3548	114,899	$5324	$163	$1500	$46
**Scenario 2: Care seeking (low: HIV 40% HIVHTN 40%); proportion of well‐controlled patients (high: HIV 90% HIVHTN 70%)**																	
Status quo	119,398	Ref	87,929	Ref	12,837	Ref	454,512	Ref	32,735	Ref	Ref	Ref	Ref	Ref	Ref	Ref	Ref
6MMD for HIV only	131,481	10%	100,559	14%	11,648	−9%	415,867	−9%	34,263	5%	12,630	1189	38,645	$1528	$121	$1286	$40
6MMD for HIV+HIVHTN	131,481	10%	100,559	14%	11,648	−9%	415,867	−9%	32,001	−2%	12,630	1189	38,645	−$733	−$58	−$617	−$19
Integrated 6MMD for HIV+HIVHTN	153,132	28%	124,217	41%	9470	−26%	345,508	−24%	35,973	10%	36,288	3367	109,005	$3238	$89	$962	$30
**Scenario 3: Care seeking (high: HIV 90% HIVHTN 80%); proportion of well‐controlled patients (low: HIV 50% HIVHTN 30%)**																	
Status quo	131,332	Ref	79,794	Ref	13,032	Ref	453,164	Ref	35,899	Ref	Ref	Ref	Ref	Ref	Ref	Ref	Ref
6MMD for HIV only	146,871	12%	94,012	18%	11,592	−11%	405,663	−10%	38,384	7%	14,218	1440	47,500	$2485	$175	$1726	$52
6MMD for HIV+HIVHTN	146,871	12%	94,012	18%	11,592	−11%	405,663	−10%	36,502	2%	14,218	1440	47,500	$603	$42	$419	$13
Integrated 6MMD for HIV+HIVHTN	175,595	34%	121,969	53%	8844	−32%	315,796	−30%	42,520	18%	42,175	4188	137,368	$6621	$157	$1581	$48
**Scenario 4: Care seeking (high: HIV 90% HIVHTN 80%); proportion of well‐controlled patients (high: HIV 90% HIVHTN 70%)**																	
Status quo	139,656	Ref	106,345	Ref	12,028	Ref	420,990	Ref	38,566	Ref	Ref	Ref	Ref	Ref	Ref	Ref	Ref
6MMD for HIV only	154,855	11%	122,292	15%	10,630	−12%	374,964	−11%	40,514	5%	15,946	1398	46,026	$1948	$122	$1394	$42
6MMD for HIV+HIVHTN	154,855	21%	122,292	15%	10,630	−12%	374,964	−11%	37,707	−2%	15,946	1398	46,026	−$859	−$54	−$614	−$19
Integrated 6MMD for HIV+HIVHTN	182,217	42%	152,326	43%	8045	−33%	290,491	−31%	42,661	11%	45,980	3983	130,500	$4095	$89	$1028	$31

Abbreviations: DALY, disability‐adjusted life year; HIVHTN, HIV‐hypertension comorbidity; MMD, multi‐month drug dispensing.

The integrated 6MMD for HIV and HIVHTN strategy resulted in incremental costs between $3.2 and $6.6 million at an additional 33,000−46,000 controlled patients and 3,400−4,200 deaths averted over 10 years, compared to the status quo, depending on the setting‐specific conditions. For the integrated 6MMD strategy, deaths/DALY averted and incremental costs were the highest when care‐seeking was high and the proportion of controlled patients was low (Scenario 3: crowded and low‐quality clinic settings) because the integrated 6MMD could offer more opportunities for improving service quality and adherence (i.e. reduce LTFU and mortality of patients in care) compared to the status quo. On the other hand, deaths/DALYs averted and incremental costs were the lowest when care‐seeking was low, and the proportion of controlled patients was high (Scenario 2: uncrowded and high‐quality clinic settings). Collectively, the most cost‐effective scenarios for integrated 6MMD were when the proportion of controlled patients was high (Scenarios 2 and 4; $30−31/DALY averted) and the least cost‐effective scenarios were when the proportion of controlled patients was low (Scenarios 1 and 3; $46−48/DALY averted).

**Figure 2:**
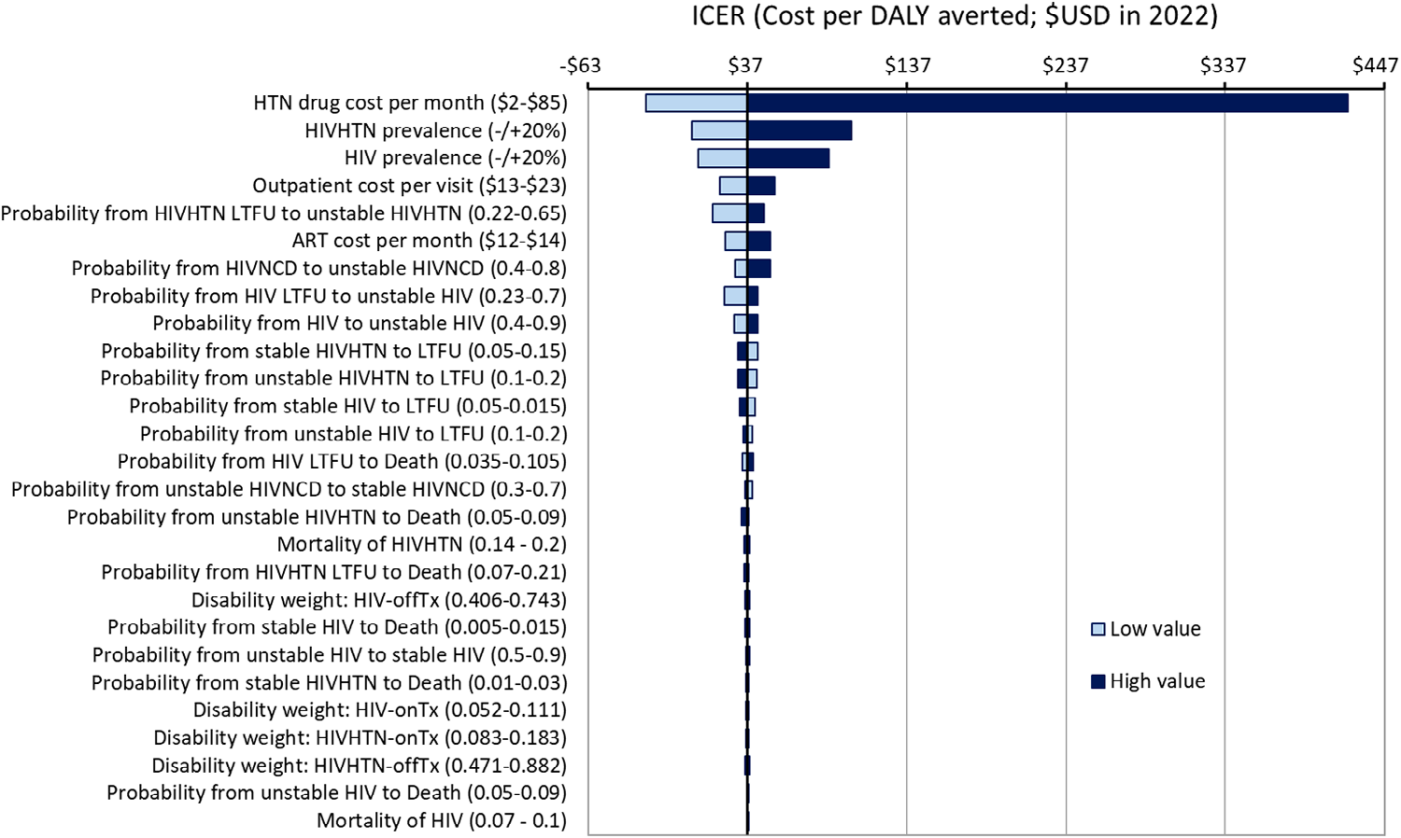
One‐way sensitivity analysis of integrated 6‐month multi‐month dispensing (6MMD) for people living with HIV only and people living with HIV and hypertension comorbidity in South Africa, compared with the status quo (without 6MMD). DALY, disability‐adjusted life year; HIVHTN, HIV‐hypertension comorbidity; LTFU, loss‐to‐follow‐up.

**Figure 3:**
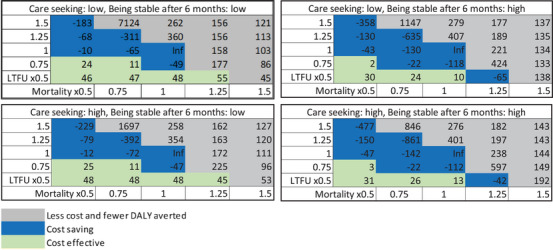
Cost‐effectiveness (incremental cost in 2022 US dollars per disability‐adjusted life year (DALY) averted) for three‐way sensitivity analyses of integrated 6‐month multi‐month dispensing (6MMD) for people living with HIV only and people living with HIV and hypertension comorbidity in South Africa, compared with a status quo of no 6MMD. LTFU, loss‐to‐follow‐up. Inf = Infinite as incremental DALY averted is 0.

Probabilistic sensitivity analyses showed that 30% of simulations were cost‐saving for integrated 6MMD strategy (about 50% for 6MMD strategies without integration) (Appendix Figure S1). Our one‐way sensitivity analysis showed that HTN drug cost and HIVHTN and HIV prevalences rate were key drivers in the cost per DALY averted (Figure 2 and Appendix Figure S2). The three‐way sensitivity analyses demonstrated that the ICER may vary substantially by the relative reduction of LTFU and mortality rate (Figure 3). When mortality rates with integrated 6MMD were greater than 1.5 times status quo values, most conditions resulted in fewer costs and fewer DALYs averted. When mortality rates with integrated 6MMD were below the 0.5 times status quo values, most conditions resulted in cost‐savings or were highly cost‐effective (being less than $50 per DALY averted) across a wide range of LTFU factor variation (−/+50% status quo values).

## 4 DISCUSSION

Under the scenarios in our model, integrated 6MMD for HIV and HTN in South Africa could avert between 0.8 and 1 DALYs and increase health systems costs between $24 and $49 per HIV or HIVHTN patient annually, translating to between $962 and $1581 per death averted and between $30 and $48 per DALY averted over 10 years. The health outcomes and health systems costs depend on care‐seeking levels, the proportion of well‐controlled patients in care and 6MMD enrolment; these variables determine the total number of patients entering and remaining in the health system, eligible to enrol in 6MMD and clinical visit frequency. Overall, integrated 6MMD achieved the greatest health outcomes and incremental costs in settings with high care‐seeking but low proportion of well‐controlled patients (i.e. crowded and low‐quality clinic settings). However, this scenario led to the least cost‐effective ICER value ($48/DALY averted), primarily because the large number of patients in care significantly increased health system costs, outweighing the associated health benefits. An increased proportion of well‐controlled patients (i.e. crowded but high‐quality clinic settings) could improve cost‐effectiveness to $31/DALY averted. Reducing mortality by half could make most scenarios cost‐saving or highly cost‐effective (under $50 per DALY averted). These findings highlight integrated 6MMD's benefits beyond reducing costs from fewer clinic visits, as it may also achieve high service quality that results in less LTFU and fewer deaths.

Our study shows that scale‐up of 6MMD strategies may be more effective or cost‐saving when combined with strategies to address multiple conditions and integration. Moreover, the consequence of 6MMD (fewer clinic visits for stable patients) at the population level depends on the setting. In our model, the proportion of care‐seeking parameters reflects potential interventions to increase service awareness and access to treatment initiation. The proportion of well‐controlled patients after 6 months may reflect an intervention that can promote better service quality and accountability to manage patients’ disease progression. The integrated 6MMD is expected to improve care‐seeking and control among patients already in care, reducing LTFU and deaths of all patients in care due to improved provider capacity. Consequently, more patients remain in the health system, increasing health benefits and costs. Counterintuitive results, such as greater cost savings with higher LTFU than lower LTFU, as shown in Figure 3, are common in cost‐effectiveness analyses where effectiveness is influenced by the level of retention in care. Despite this, our results indicate that across a wide range of LTFU factor variation (−/+50% status quo), integrated MMD can achieve cost savings or is highly cost‐effective if it maintains or reduces the mortality rates of the patients in the care, compared to the status quo values.

A few studies using microsimulation or decision tree models have evaluated integrated screening for HIV and NCD to prevent cardiovascular diseases (CVDs), reporting cost‐effectiveness at $860 per DALY averted in Kenya [50] or $1400−$3250 per DALY averted in Uganda [51]. Based on the Framining calculator [52] or the Globorisk mathematical model [53], these risk prediction model studies assessed the impact of integrated screening among HIV patients to identify high‐risk groups (HIV/HTN comorbid patients) and estimate the prevention of future CVD risks. In contrast, our study examined the impact of the integrated treatment, focusing on reducing clinic visits for drug dispensing. This approach aims to save service provision time for providers, improve service quality and reduce LTFU for patients, as this has not been previously explored. A key strength of our study is its consideration of varying demand and supply conditions that influence service volume, LTFU and mortality over time. This allows us to demonstrate the incremental benefit of service integration and 6MMD for stable patients, including its impact on total programme cost and health outcomes of all HIV and HIVHTN patients.

Our study has some limitations. First, we used simplifying assumptions that may not fully capture the complex interaction between HIV and HTN (e.g. heterogeneous mixing and varying screening or testing management within risk groups). For example, as we focused on assessing the incremental value of integrated 6MMD to HIV and HIVHTN patients from the deterministic model, we did not consider newly acquired HIV or HTN cases or changing HIV or HYP incidence rates over time. We also ignored the annual incidence rate of 0.054 [5] of hypertension among HIV patients over 10 years in this model, as it had minimal impact on health outcomes (<5%) and programme costs (<0.1%). While HIV incidence may decline and HTN incidence rises over time, we assumed that these changes were reflected in care‐seeking variations in our sensitivity analyses. Second, we applied constant transition rates across all age groups due to limited data, though older groups may have higher progression and LTFU/mortality rates. Age‐specific outcomes in our model are mainly driven by HIV and HIVHTN prevalence and life expectancy for DALY estimation (Appendix Table S2 and Figure S3). Incorporating higher transition rates for older patients could improve health outcomes, but as our ICER values focus on the incremental value of 6MMD and integration compared to the status quo, the balance between increased service volume and costs would not alter our conclusion that integrated 6MMD is highly cost‐effective. A recent cross‐sectional study in rural KwaZulu‐Natal, South Africa, identified age‐specific met and unmet health needs for HIV, hypertension and diabetes [45, 54]. Our model, defined by care‐seeking and well‐controlled patient proportions, aligns with these categorized needs gaps (Needs score 1−4) and accounts for regional and operational heterogeneity. Future studies may incorporate age‐specific estimates and additional comorbidities like diabetes to improve targeting strategies. Third, while our model focused on medication dispensing for HIV or HTN treatment after screening, integrated HTN screening for new or existing HIV patients may require different management or more clinic visits for testing until their disease condition is controlled. Although Scenarios 2 and 4 yielded similar ICERs, higher care‐seeking (Scenario 4) could incur greater initial costs of screening and management, reducing cost‐effectiveness than the current estimate. HTN treatment costs may also exceed the current estimates if costs associated with future CVD events are included. We addressed such uncertainty through sensitivity analyses, reflected in the wide HTN treatment monthly cost range ($2–$85). Finally, fewer clinic visits for stable patients may require coordinated service delivery and innovative monitoring systems (e.g. digital health) to maintain retention and health outcomes, potentially requiring additional resources.

Leveraging existing HIV infrastructure to introduce HTN care is likely cost‐effective and can strengthen the health system's capacity to address the growing HTN epidemic. However, expected NCD care benefits from HIV care may be limited in settings facing challenges like overburdened staff, low motivation, drug stock‐outs and long patient wait times [55, 56]. Further evidence is needed on the impact of integration on patients not yet linked to care, those already in care and those who have dropped out. Health services research should explore economies of scope (i.e. level of service integration) and scale (i.e. higher service volume with improved care‐seeking and quality) alongside resource or system requirements such as screening protocols and workforce management, to effectively integrate HTN into HIV care in sub‐Saharan Africa.

## 5 CONCLUSIONS

In summary, we have developed a model to conceptualize and quantify expected service demand under various supply and demand conditions and uncertainties of the key parameters to determine the value of integrated 6MMD for the treatment of patients with HIV and NCDs. Our study demonstrated that scaling up 6MMD strategies may be more effective when multiple conditions are addressed simultaneously. This study also suggests that the benefits of integrated 6MMD may be greater than solely the reduced costs from fewer clinic visits, as 6MMD may also achieve less LTFU and fewer deaths.

## COMPETING INTERESTS

The authors declare no competing interests.

## AUTHORS’ CONTRIBUTIONS

RH was the mentor on the grant that funded this work. YJ and RH conceptualized the analytical framework and developed epidemiological models and scenarios. YJ performed the modelling and economic analyses and drafted the manuscript. YJ and RH accessed and verified all data. SR, BEN, LJ and NL provided key information regarding the implementation to inform cost and epidemiological assumptions and reviewed scenarios. All authors aided in interpreting the results and worked on the manuscript. All authors critically reviewed and approved the final manuscript and shared final responsibility for the decision to submit it for publication.

## FUNDING

YJ is supported by the National Institute of Mental Health (grant number F32 MH128120).

## DISCLAIMER

The content is solely the responsibility of the authors and does not necessarily represent the official views of the National Institutes of Health.

## Supporting information



Supporting Information

**Table S1**. Key input parameters
**Table S2**. Age‐specific number of patients, health outcomes and health systems costs by scenarios (2022–2031)
**Figure S1**. Cost‐effectiveness plane and cost‐effectiveness acceptability curves by intervention scenarios compared to the status quo
**Figure S2**. One‐way sensitivity analysis of integrated 6‐month multi‐month dispensing (6MMD) for people living with HIV only compared to the status quo (panel A) and 6MMD for people living with HIV and hypertension comorbidity (panel B) in South Africa, compared with the status quo (without 6MMD). The dashed grey line indicates the cost‐saving threshold.
**Figure S3**. Age group specific cost‐effectiveness plane and cost‐effectiveness acceptability curves of integrated 6MMD to both HIV and HIVHTN compared to the status quo

## Data Availability

The data that support the findings of this study are available from the published references, as indicated in Table 1.
