# Supplementary material for: Integrated multi‐month dispensing for HIV and hypertension in South Africa: A model of epidemiological impact and cost‐effectiveness
Source: J Int AIDS Soc. 2025 Feb 12;28(2):e26413. doi: 10.1002/jia2.26413 (PMC12020916; doi:10.1002/jia2.26413)
Supplement: Supplementary file 2 — Table S1. Key input parameters Table S2. Age‐specific number of patients, health outcomes and health systems costs by scenarios (2022–2031) Figure S1. Cost‐effectiveness plane and cost‐effectiveness acceptability curves by intervention scenarios compared to the status quo Figure S2. One‐way sensitivity analysis of integrated 6‐month multi‐month dispensing (6MMD) for people living with HIV only compared to the status quo (panel A) and 6MMD for people living with HIV and hypertension comorbidity (panel B) in South Africa, compared with the status quo (without 6MMD). The dashed grey line indicates the cost‐saving threshold. Figure S3. Age group specific cost‐effectiveness plane and cost‐effectiveness acceptability curves of integrated 6MMD to both HIV and HIVHTN compared to the status quo [file JIA2-28-e26413-s001.docx]

**Appendix.**

Table S1. Key input parameters

| **Parameter Description** | | **Model Value** | |  |  |  |  |  |  |
| --- | --- | --- | --- | --- | --- | --- | --- | --- | --- |
| Demographic parameters | | Male | Female |  | | | | | |
| Population | Age 0-14 | 14,399 (30%) | 13,900 (27%) | Estimated from age distribution based on a hypothetical 100,000 general population in South Africa | | | | | |
|  | Age 15-24 | 8,063 (17%) | 7,799 (15%) |  |  |  |  |  |  |
|  | Age 25-44 | 16,715 (34%) | 16,469 (32%) |  |  |  |  |  |  |
|  | Age 45-64 | 7,622 (16%) | 9,180 (18%) |  |  |  |  |  |  |
|  | Age 65+ | 1,950 (4%) | 3,902 (8%) |  |  |  |  |  |  |
| Target population | | Male | Female |  | | | | | |
| HIV | Age 15-24 | 268 (10%) | 487 (10%) | Sensitivity analyses varied the HIV initial prevalence +/-20% | | | | | |
|  | Age 25-44 | 2,010 (72%) | 3,584 (72%) |  |  |  |  |  |  |
|  | Age 45-64 | 480 (17%) | 877 (18%) |  |  |  |  |  |  |
|  | Age 65+ | 23 (1%) | 58 (1%) |  |  |  |  |  |  |
| HIVHTN | Age 15-24 | 115 (5%) | 325 (9%) |  |  |  |  |  |  |
|  | Age 25-44 | 1,184 (53%) | 2,077 (58%) |  |  |  |  |  |  |
|  | Age 45-64 | 880 (39%) | 970 (27%) |  |  |  |  |  |  |
|  | Age 65+ | 69 (3%) | 231 (6%) |  |  |  |  |  |  |
|  |  | Base case (mean) | | Low | High | SD | Shape | Scale | Distribution |
| HIV | Probability of untreated HIV to Death | 0.08 | | 0.07 | 0.1 | 0.02 | 25 | 289 | Beta |
|  | Probability from HIV positive to HIV uncontrolled (care-seeking/linkage to care) | 0.6 | | 0.4 | 0.9 | 0.25 | 2 | 1 | Beta |
|  | Probability from HIV uncontrolled to HIV controlled (6MMD enroll) | 0.7 | | 0.5 | 0.9 | 0.20 | 3 | 1 | Beta |
|  | Probability from HIV uncontrolled to LTFU^§^ | Status quo: 0.3 | | 0.150 | 0.450 | 0.15 | 3 | 6 | Beta |
|  |  | Any 6MMD/Integration: 0.24 | | 0.1 | 0.3 | 0.12 | 3 | 9 | Beta |
|  |  | All 6MMD+Integration: 0.15 | | 0.1 | 0.2 | 0.08 | 3 | 18 | Beta |
|  | Probability from HIV controlled to LTFU^§^ | Status quo: 0.2 | | 0.1 | 0.3 | 0.10 | 3 | 12 | Beta |
|  |  | Any 6MMD/Integration: 0.16 | | 0.08 | 0.24 | 0.08 | 3 | 17 | Beta |
|  |  | All 6MMD+Integration: 0.1 | | 0.05 | 0.15 | 0.08 | 3 | 18 | Beta |
|  | Probability from HIV uncontrolled to Death^†^ | Base case: 0.06 | | 0.05 | 0.09 | 0.02 | 8 | 121 | Beta |
|  |  | Any 6MMD/Integration: 0.048 | | 0.04 | 0.07 | 0.02 | 8 | 156 | Beta |
|  |  | All 6MMD+Integration:0.03 | | 0.025 | 0.045 | 0.01 | 8 | 260 | Beta |
|  | Probability from HIV controlled to Death^†^ | Base case: 0.02 | | 0.01 | 0.03 | 0.02 | 4 | 191 | Beta |
|  |  | Any 6MMD/Integration: 0.016 | | 0.008 | 0.024 | 0.01 | 4 | 241 | Beta |
|  |  | All 6MMD+Integration: 0.01 | | 0.005 | 0.015 | 0.01 | 4 | 391 | Beta |
|  | Probability from HIV_LTFU to Death | 0.07 | | 0.035 | 0.105 | 0.04 | 4 | 48 | Beta |
|  | Probability from HIV_LTFU to HIV uncontrolled (re-enrollment) | 0.465 | | 0.23 | 0.7 | 0.23 | 2 | 2 | Beta |
| HIVHTN | Probability of untreated HIVHTN to Death | 0.16 | | 0.14 | 0.2 | 0.03 | 23 | 120 | Beta |
|  | Probability from HIVHTN positive to HIVHTN uncontrolled (care-seeking/linkage to care) | 0.5 | | 0.4 | 0.8 | 0.21 | 2 | 2 | Beta |
|  | Probability from HIVHTN uncontrolled to HIVHTN controlled (6MMD enroll) | 0.5 | | 0.3 | 0.7 | 0.20 | 3 | 3 | Beta |
|  | Probability from HIVHTN uncontrolled to LTFU^§^ | Status quo: 0.3 | | 0.150 | 0.450 | 0.15 | 3 | 6 | Beta |
|  |  | Any 6MMD/Integration: 0.24 | | 0.1 | 0.3 | 0.12 | 3 | 9 | Beta |
|  |  | All 6MMD+Integration: 0.15 | | 0.1 | 0.2 | 0.08 | 3 | 18 | Beta |
|  | Probability from HIVHTN controlled to LTFU^§^ | Status quo: 0.2 | | 0.100 | 0.300 | 0.10 | 3 | 12 | Beta |
|  |  | Any 6MMD/Integration: 0.16 | | 0.08 | 0.24 | 0.08 | 3 | 17 | Beta |
|  |  | All 6MMD+Integration: 0.1 | | 0.05 | 0.15 | 0.08 | 3 | 18 | Beta |
|  | Probability from HIVHTN uncontrolled to Death^†^ | Base case: 0.12 | | 0.1 | 0.18 | 0.04 | 7 | 53 | Beta |
|  |  | 6MMD/Integration: 0.1 | | 0.1 | 0.14 | 0.03 | 12 | 110 | Beta |
|  |  | All 6MMD+Integration: 0.06 | | 0.08 | 0.09 | 0.02 | 14 | 226 | Beta |
|  | Probability from HIVHTN controlled to Death^†^ | Base case: 0.04 | | 0.04 | 0.06 | 0.02 | 4 | 91 | Beta |
|  |  | 6MMD/Integration: 0.032 | | 0.016 | 0.05 | 0.03 | 4 | 116 | Beta |
|  |  | All 6MMD+Integration: 0.02 | | 0.01 | 0.03 | 0.02 | 4 | 191 | Beta |
|  | Probability from HIVHTN LTFU to Death (mortality) | 0.14 | | 0.07 | 0.21 | 0.07 | 3 | 20 | Beta |
|  | Probability from HIVHTN_LTFU to HIVHTN uncontrolled (re-enrollment) | 0.43 | | 0.215 | 0.645 | 0.22 | 2 | 2 | Beta |
| Disability weight for DALYs | |  | |  |  |  |  |  |  |
| HIV on Treatment | | 0.078 | | 0.052 | 0.111 | 0.03 | 6.96 | 0.01 | Triangular |
| HIV off Treatment | | 0.582 | | 0.406 | 0.743 | 0.17 | 11.92 | 0.05 | Triangular |
| HIVHTN -on Treatment | | 0.127 | | 0.083 | 0.183 | 0.05 | 6.42 | 0.02 | Triangular |
| HIVHTN -off Treatment | | 0.679 | | 0.471 | 0.882 | 0.21 | 10.92 | 0.06 | Triangular |
| Cost parameters | |  | |  |  |  |  |  |  |
| ART cost per person per month | | $13 | | $12 | $14 | 1.00 | 169 | 0.08 | Gamma |
| HTN drug cost per person per month | | $14 | | $2 | $85 | 6.70 | 4.37 | 3.20 | Gamma |
| Outpatient cost per person per visit | | $18 | | $13 | $23 | 6.43 | 12.96 | 1.39 | Gamma |

Figure S1. Cost-effectiveness plane and cost-effectiveness acceptability curves by intervention scenarios compared to the status quo

| 1. Cost-effectiveness plane | 1. Cost-effectiveness acceptability curve |
| --- | --- |
| 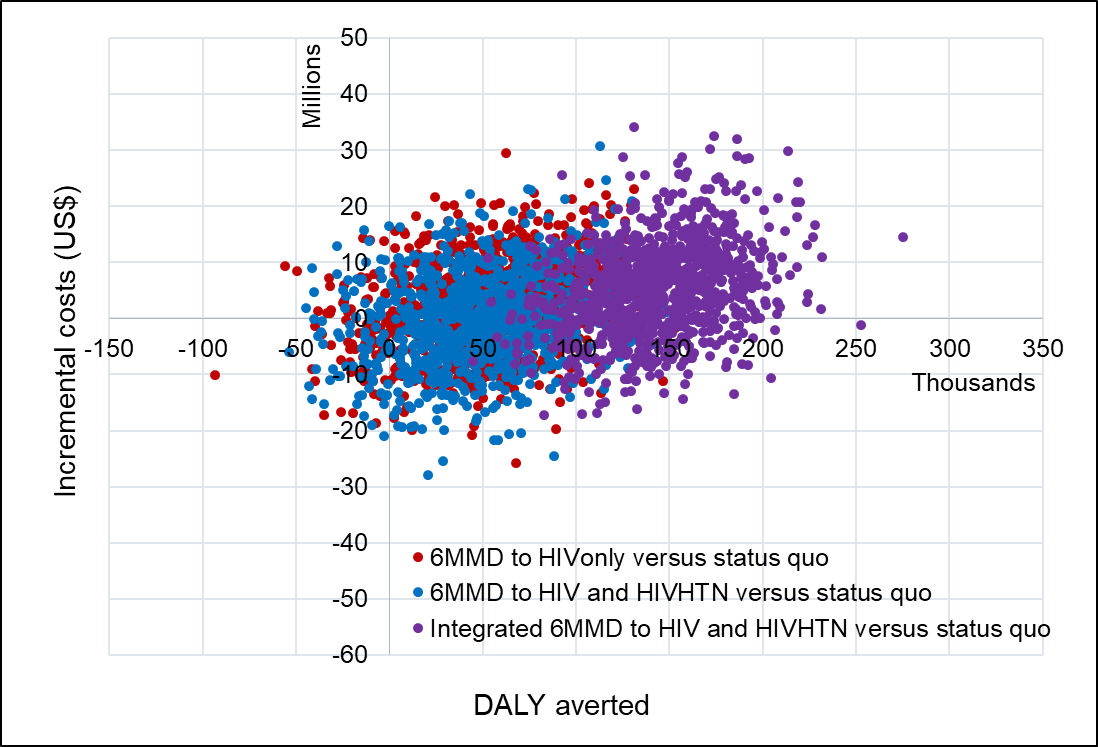 | 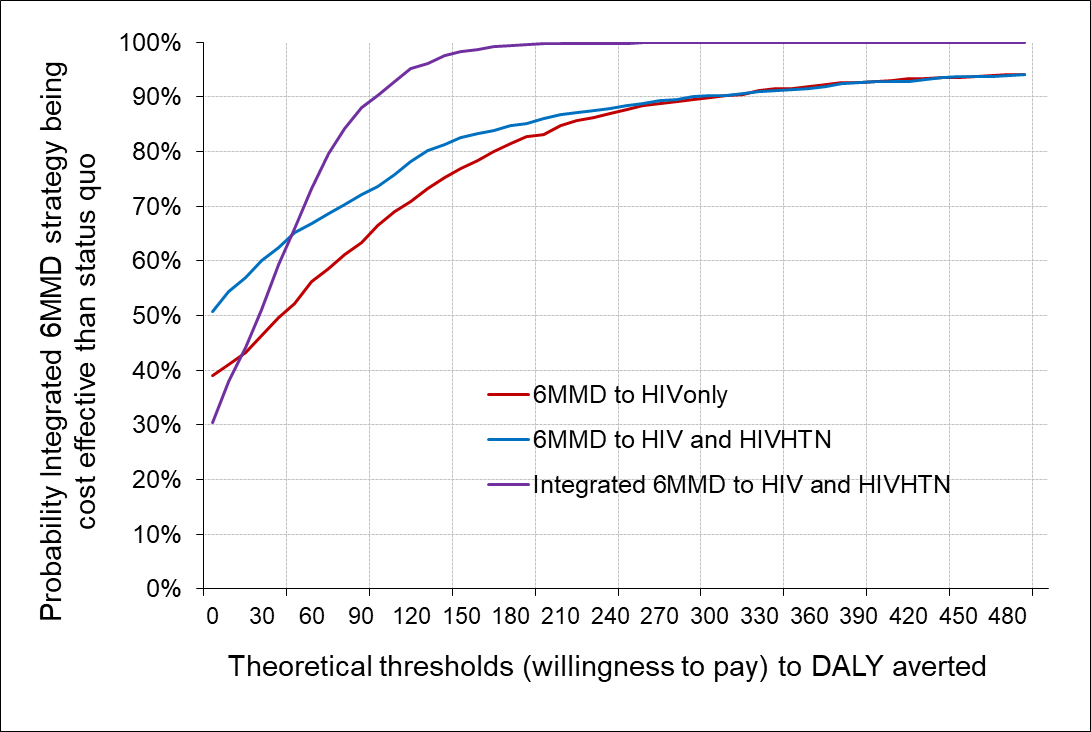 |

Figure S2. One-way sensitivity analysis of integrated 6-month multi-month dispensing (6MMD) for people living with HIV only compared to the status quo (panel A) and 6MMD for people living with HIV and hypertension comorbidity (panel B) in South Africa, compared with the status quo (without 6MMD). The dashed grey line indicates the cost-saving threshold.

1. 6MMD to HIV only compared to status quo


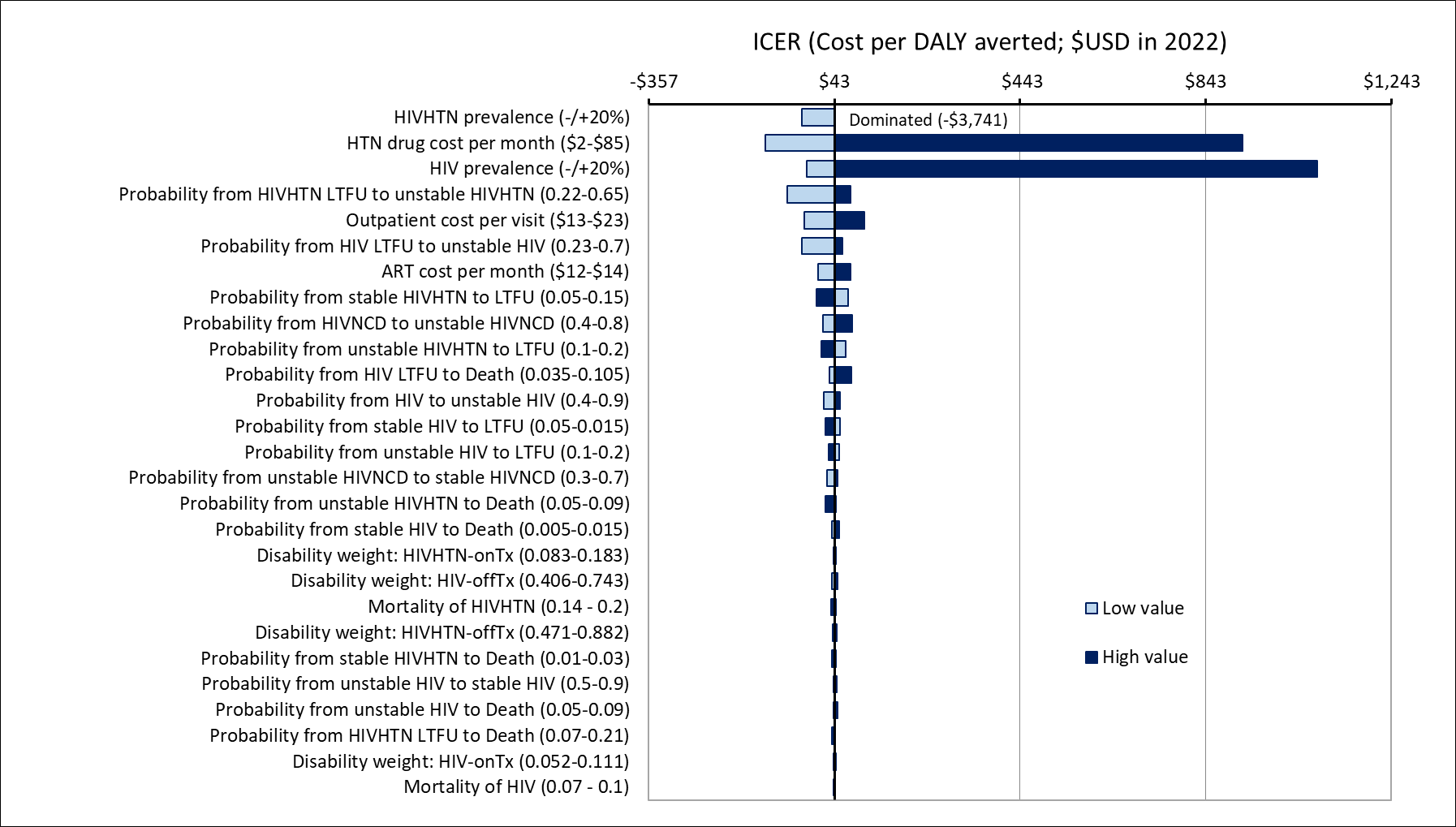


1. 6MMD to HIV and HIVHTN compared to status quo


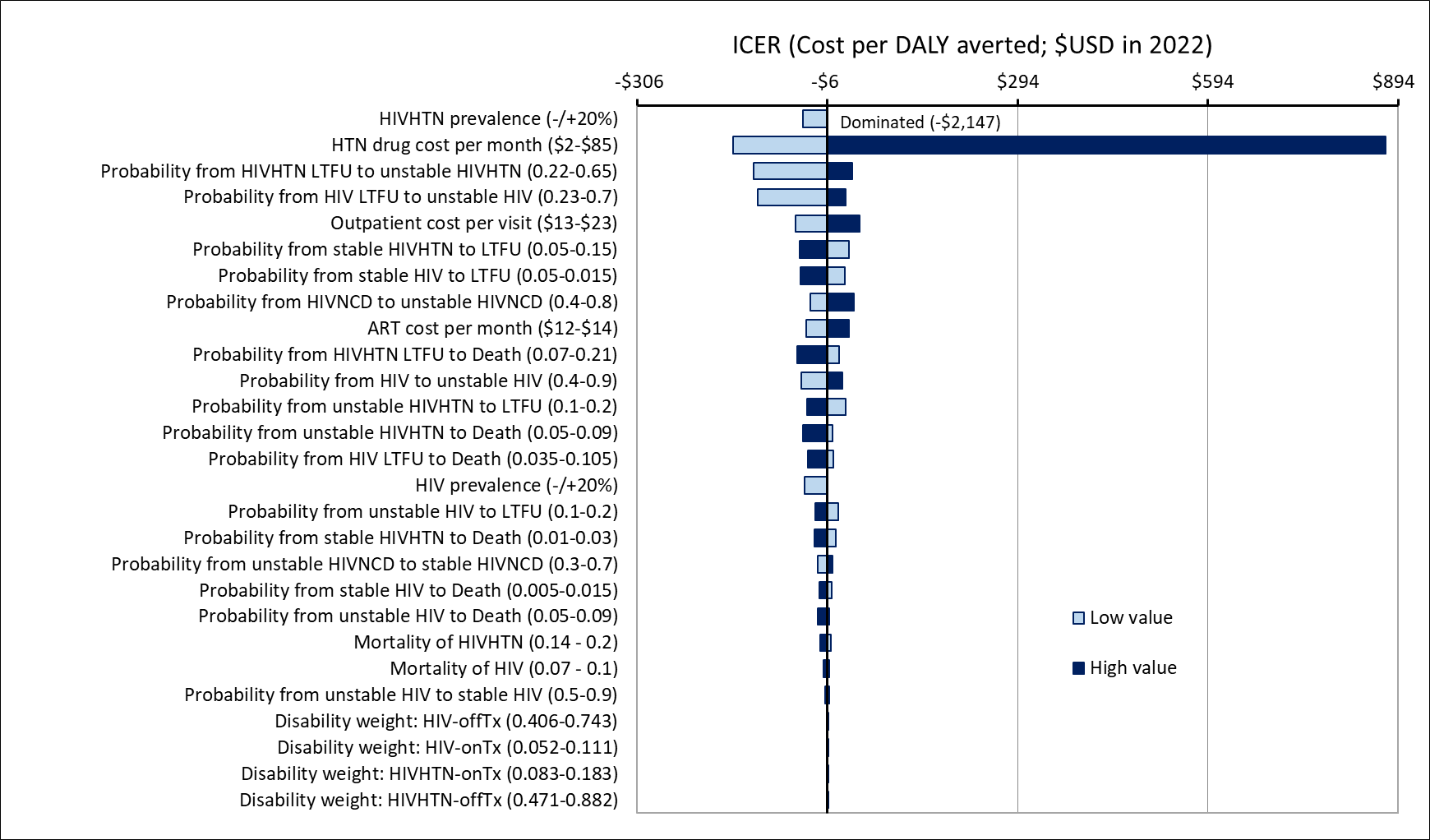


Table S2. Age-specific number of patients, health outcomes and health systems costs by scenarios (2022-2031)

Age-specific results showed that the optimal (most cost-effective) strategy may differ by age group and setting specific conditions. Among the younger age group (age 15-24), 6MMD without integration were cost saving or more cost effective than alterhative strategies in general; among the older age group (age ≥ 65) however, implementing integrated 6MMD to both HIV and HIVHTN was always the most cost effective strategy than alternative strategies across various settings. Comparing the ICER values across age groups, the integrated 6MMD for HIV and HIVHTN was relatively higher among the older age groups than the younger age groups (i.e., from $25 to $133 per DALY averted for age < 15 and age ≥ 65 groups, respectively, for the base case scenario). This was due to the larger prevalence of HIV and HTN in older age groups that led to greater service volume which increased total cost far greater than increased total health benefits. 6MMD to both HIV and HIVHTN without integration led to cost saving compared to the status quo in most scenarios because the reduced outpatient visit costs far exceeded the increased drug costs of patients who initiate and remain in care.

In terms of considering targeting strategy, if we consider HIV patients aged older than 45 as a high risk group, this patients group consists of about 30% of total number of people living with HIV. The reduced visit frequency by targeting the older patients may have a marginal impact on improving provider capacity and overall service quality and our thus assumption about the effectiveness of 6MMD (20/50% reduced LTFU/mortality of all patients) may be too optimistic. If the targeted integrated 6MMD to older patients results in similar health outcomes like the status quo for all patients but only reduces the clinic visit cost for the well controlled old patients, it will be “cost saving” compared to the status quo. While targeting a high-risk subgroup (older patients) may have less benefit on the overall health outcome of all patients (compared to delivering integrated 6MMD to stable patients of all ages), this could be a more feasible and efficient approach under cost and workforce capacity constraints.

Figure S3. Age group specific cost-effectiveness plane and cost-effectiveness acceptability curves of integrated 6MMD to both HIV and HIVHTN compared to the status quo

| 1. Cost-effectiveness plane | 1. Cost-effectiveness acceptability curve |
| --- | --- |
| 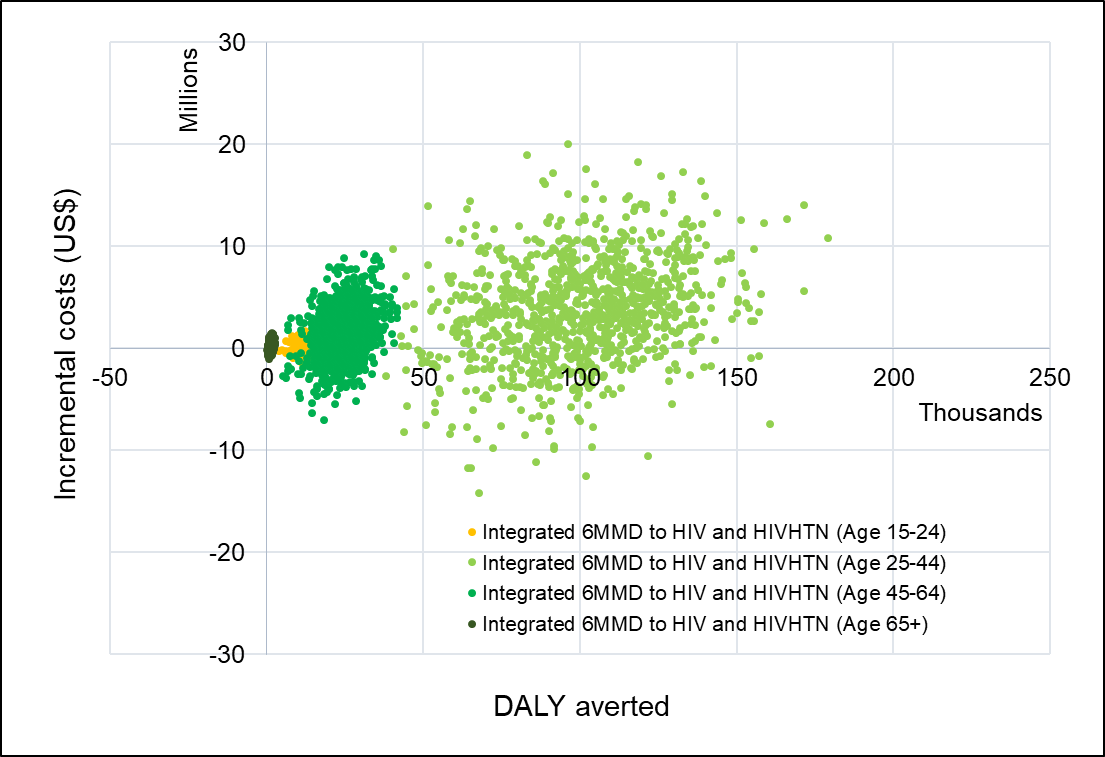 | 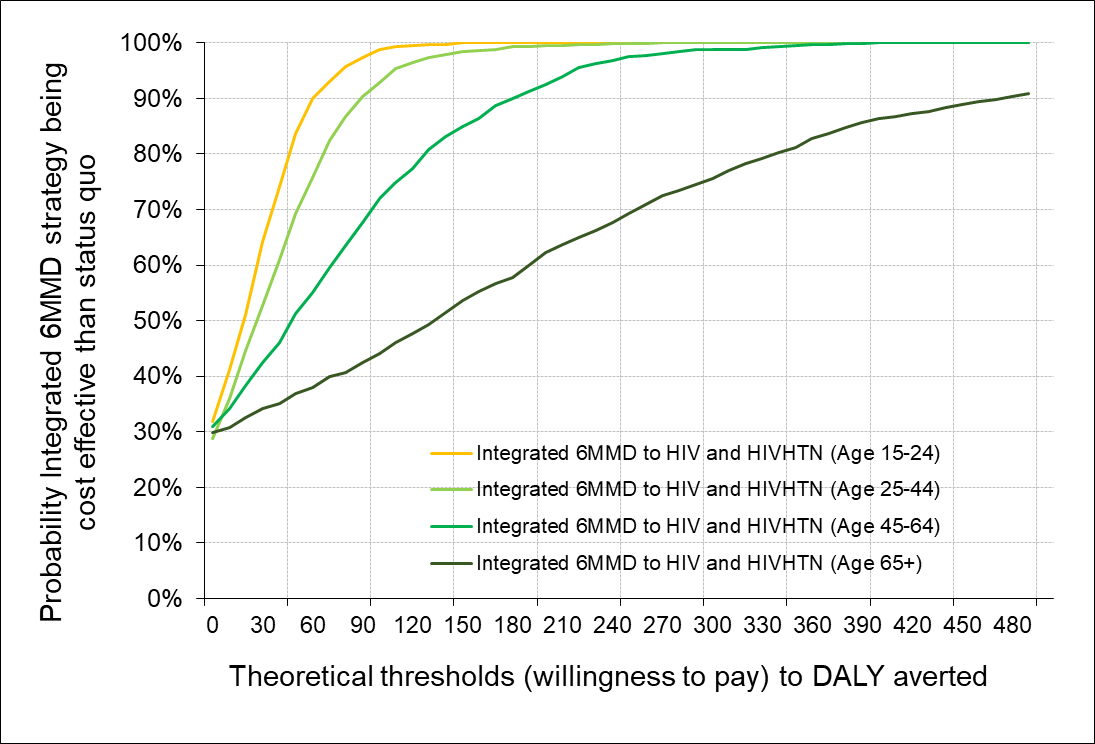 |
